# Bovine Intelectin 2 Expression as a Biomarker of Paratuberculosis Disease Progression

**DOI:** 10.3390/ani11051370

**Published:** 2021-05-12

**Authors:** Cristina Blanco Vázquez, Ana Balseiro, Marta Alonso-Hearn, Ramón A. Juste, Natalia Iglesias, Maria Canive, Rosa Casais

**Affiliations:** 1 Center for Animal Biotechnology, Servicio Regional de Investigación y Desarrollo Agroalimentario (SERIDA), 33394 Deva, Spain; cristina.blancovazquez@serida.org (C.B.V.); nibesteiro@serida.org (N.I.); 2 Departamento de Sanidad Animal, Facultad de Veterinaria, Universidad de León, 24071 León, Spain; abalm@unileon.es; 3 Departamento de Sanidad Animal, Instituto de Ganadería de Montaña (CSIC-Universidad de León), Finca Marzanas, Grulleros, 24346 León, Spain; 4 Department of Animal Health, NEIKER-Basque Institute for Agricultural Research and Development, Basque Research and Technology Alliance (BRTA), Parque Científico y Tecnológico de Bizkaia, P812, E-48160 Derio, Spain; malonso@neiker.eus (M.A.-H.); rjuste@neiker.eus (R.A.J.); mcanive@neiker.eus (M.C.)

**Keywords:** intelectin 2, biomarker, immunohistochemistry, ileocecal valve, *Mycobacterium* *avium* subsp. *paratuberculosis*, paratuberculosis

## Abstract

**Simple Summary:**

The potential of the bovine intelectin 2 as a biomarker of *Mycobacterium* *avium* subsp. *paratuberculosis* infection was investigated using quantitative immunohistochemical analysis of ileocecal valve samples of animals with increasing degrees of lesion severity (focal, multifocal and diffuse histological lesions) and control animals without detected lesions. Significant differences were observed in the mean number of intelectin 2 immunolabelled cells between the three histopathological types and the control. Specifically, the mean number of intelectin 2 labelled cells was indicative of disease progression as the focal group had the highest number of intelectin 2 secreting cells followed by the multifocal, diffuse and control groups indicating that intelectin 2 is a good biomarker for the different stages of *Mycobacterium* *avium* subsp. p*aratuberculosis* infection. Quantification of bovine intelectin 2 secreting cells could constitute a good post-mortem tool, complementary to histopathology, to improve detection of *Mycobacterium* *avium* subsp. *Paratuberculosis* infections, especially latent forms of infection.

**Abstract:**

Paratuberculosis (PTB), a chronic granulomatous enteritis caused by *Mycobacterium* *avium* subsp. *paratuberculosis* (MAP), is responsible for important economic losses in the dairy industry. Our previous RNA-sequencing (RNA-Seq) analysis showed that bovine intelectin 2 (ITLN2) precursor gene was overexpressed in ileocecal valve (ICV) samples of animals with focal (log2 fold-change = 10.6) and diffuse (log2 fold-change = 6.8) PTB-associated lesions compared to animals without lesions. This study analyzes the potential use of ITLN2, a protein that has been described as fundamental in the innate immune response to infections, as a biomarker of MAP infection. The presence of ITLN2 was investigated by quantitative immunohistochemical analysis of ICV samples of 20 Holstein Friesian cows showing focal (*n* = 5), multifocal (*n* = 5), diffuse (*n* = 5) and no histological lesions (*n* = 5). Significant differences were observed in the mean number of ITLN2 immunostained goblet and Paneth cells between the three histopathological types and the control. The number of immunolabelled cells was higher in the focal histopathological type (116.9 ± 113.9) followed by the multifocal (108.7 ± 140.5), diffuse (76.5 ± 97.8) and control types (41.0 ± 81.3). These results validate ITLN2 as a post-mortem biomarker of disease progression.

## 1. Introduction

Bovine paratuberculosis (PTB) or Johne’s disease, is a chronic granulomatous enteritis of ruminants responsible for important economic losses in dairy herds worldwide due to reduced milk production, premature culling and reduced slaughter value [1,2]. PTB is caused by *Mycobacterium avium* subsp. *paratuberculosis* (MAP), a mycobacteria with zoonotic potential since it has been postulated as a possible trigger factor in several autoimmune diseases in humans such as Crohn’s disease [3,4], rheumatoid arthritis [5,6], multiple sclerosis [7,8] or type I diabetes [9].

Two forms of infection, latent and patent, can be defined in MAP infected cattle. The disease is typically present as a subclinical highly prevalent latent form of MAP infection defined by focal granulomatous lesions in the intestine and associated lymph nodes, low bacterial load and a predominant cell-mediated immune response. The patent form of the disease, less frequent, it is characterized by the manifestation of clinical signs, multifocal and diffuse histological lesions, a higher mycobacterial load and a predominant humoral immune response with high levels of anti-MAP antibodies detected [10]. PTB control programs are currently based on testing and culling test-positive cows combined with good management practices including appropriate hygienic-sanitary strategies [11,12,13]. These control programs are strongly conditioned by the low sensitivities and specificities for the detection of latent forms of infection because bacteria are excreted in low numbers and animals have low titers of specific antibodies [14,15,16,17,18]. Consequently, novel diagnostic tools with high sensitivity and specificity are needed to improve the diagnostic and control of the disease. Emerging -omic technologies have been used for the identification of host biomarkers [19,20,21,22,23,24,25,26,27,28,29,30] that could help to understand the factors that determine disease progression from the latent to the patent form of infection and could be used in biomarker-based diagnostic methods for PTB. However, most of these biomarkers have not yet been applied and validated for PTB diagnosis in naturally infected cattle.

Using RNA-Seq analysis of peripheral blood and ileocecal valve (ICV) samples, collected from animals with focal or diffuse lesions in their intestinal tissues and control animals without detected lesions, our research group identified several biomarkers with the potential for implementation as diagnostic tools to reliably detect MAP-infection [31]. The diagnostic potential of commercial ELISAs based on the detection of five of those blood candidate biomarkers to detect latent and patent forms of the disease in naturally infected cows was evaluated. It was shown that serum quantification of ATP binding cassette subfamily A member 13 (ABCA13), secreted acidic and cysteine rich protein (SPARC) and matrix metallopeptidase 8 (MMP8) by ELISA has the potential to be used as a diagnostic tool for PTB, greatly improving early detection of MAP latent infections, which are currently escaping detection with conventional diagnostic methods [32]. In the ICV samples, the gene encoding for bovine protein intelectin 2 precursor (ITLN2) showed significantly higher levels of expression in ICV samples of cows with focal and diffuse histological lesions than in control cows with no lesions. Specifically, the ITLN2 precursor was the most upregulated gene in the ICV samples of cows with focal or diffuse lesions in comparison to the control type, with a log2 fold-change of 10.6 and 6.8, respectively [31].

Intelectins are calcium-dependent galactose binding proteins that have a variety of possible functions including alteration of mucus viscosity by binding mucine-like glycoproteins [33], protective antibacterial role via recognition of galactofuranose-containing components in bacterial cell walls leading to cell lysis [34,35] and phagocyte activation [36]. mRNA expression of intelectins increases in response to infections [37,38,39,40,41] and asthma [42], which suggests that intelectins play an important role in innate immune response to pathogens [34,37,43,44,45,46,47].

Intelectin family members have been identified in many species including cephalochordates, fish, amphibians and mammals [36,39,43,46,47,48,49,50,51,52,53]. Expression patterns of intelectin family members vary among species. For example, human intelectin 1 (hITLN1) is expressed in the heart, small intestine, colon, kidney collecting tubule cells and bladder umbrella cells, some mesothelial and follicular cells [43,54,55] whereas human intelectin 2 (hITLN2) is only found in small intestine. In mouse, intelectin 1 (mITLN1) is largely restricted to intestine; while mouse intelectin 2 (mITLN2) is detectable only in ileum [37]. In intestinal epithelium, intelectins are expressed by Paneth [48] and goblet cells [37], and have also been found within the epithelial brush border [56].

Specifically, ITLN2 (also known as intestinal lactoferrin receptor) is a Ca2β-dependent and D-galactosyl-specific lectin that is involved in host-pathogen interactions [54], iron metabolism [46] and allergic inflammation [57]. It is produced mainly in the Paneth and goblet cells [37,58]. In a previous study in mice infected with *Trichinella spiralis* [59], ITLN2 was markedly up-regulated in response to infection being one of the most abundantly expressed proteins in jejunal infected epithelium that suggested that it may play an important role in the innate immune response to parasite infection.

In a previous study, we investigated the localization of ITLN2 in ICV of animals with different types of PTB-associated histological types in their gut tissues by immunohistochemistry using a specific anti-ITLN2 antibody [31]. Intensive ITLN2 labelling of goblet and Paneth cells was observed in the ICV of infected animals compared to non-infected control samples [31]. However, the number of animals that were analyzed was very small and we did not quantify the number of specifically labelled cells.

The aim of the present study was to validate our previous RNASeq results investigating the potential of ITLN2 as a post-mortem biomarker of disease progression. A total of 20 Holstein Friesian cows, with known infection status, were classified by histopathological analysis as focal (*n* = 5), multifocal (*n* = 5), diffuse (*n* = 5) and with no histological lesions detected (*n* = 5). ITLN2 expression was evaluated by quantitative immunohistochemical analysis of ICV samples of naturally infected animals with latent (focal lesions) and patent (multifocal and diffuse lesions) MAP infections and non-infected control animals. Histopathological analysis represents the best post-mortem diagnosis method to confirm PTB and describe the form of infection present in the animal. Quantification of bovine ITLN2 secreting cells by immunohistochemical analysis of ileocecal valve sections could constitute a good post-mortem tool, complementary to histopathology, to improve detection of MAP infections. In particular, detection of latent infections in animals with focal lesions, which may be sometimes difficult to detect when the animal has low numbers or sparsely distributed granulomas, yet these animals would show numerous ITLN2 labelled cells.

## 2. Materials and Methods

### 2.1. Ethical Statement

Experimental procedures were approved by the SERIDA Animal Ethics Committee and authorised by the Regional Consejería de Agroganadería y Recursos Autóctonos del Principado de Asturias, Spain (authorization codes PROAE 29/2015 and PROAE 66/2019). All the procedures were carried out in accordance with Directive 2012/63/EU of the European Parliament.

### 2.2. Animals and Samples

Samples of blood, feces and tissues, collected from 20 Holstein Friesian cows (ranging from 0.81 to 10.39 years of age) coming from three farms located in the Principality of Asturias (Northwest of Spain), were used in this study. Blood and feces samples were taken at the farms while tissue samples (distal jejunum, ICV, and ileocecal and jejunal lymph nodes) were taken from the slaughtered animals *in situ* at the local abattoir after evisceration. Some of these 20 animals were included in a previous study [32].

### 2.3. Serological, Bacteriological and Molecular Characterization of the Animals

The PTB infection status of the 20 animals (range from 0.81 to 10.39 years) used in this study at the time of slaughter was determined by histopathology, specific antibody serum ELISA test (IDEXX, Montpellier, France), and bacteriological culture and specific real-time polymerase chain reaction (PCR) of tissues and feces following the procedures previously described [32] (Table 1). Animals were classified in four groups of five individuals by histological analysis: focal, multifocal, diffuse and control group without lesions detected. All control animals were negative for all the diagnostic tests. The rest of the animals considered as infected were positive at least by one of the diagnostic methods used. No gross or histologic lesions compatible with other inflammatory processes were identify during post-mortem inspection.

### 2.4. Histopathological Classification of Animals

For histological processing, tissue samples (distal jejunum, ICV, and ileal and jejunal lymph nodes for each cow) were fixed in 10% neutral buffered formalin, sliced and embedded in paraffin wax using standard procedures. Afterwards, 4 μm sections were stained by the haematoxylin-eosin (HE) and Ziehl-Neelsen (ZN) methods for lesion assessment and specific acid-fast bacteria (AFB) detection. The stained sections were observed with an Olympus BH-2 light microscope (Olympus, Tokyo, Japan) and lesions were classified in four types: focal, multifocal, diffuse and without lesions according to González et al. [60]. Briefly, the focal lesions consist of one or two isolated granulomas, mainly located in the jejunal and ileal lymph nodes, but also in the follicular area of the ICV and not affecting the intestinal lamina propria. The multifocal lesions consist of numerous well-demarcated granulomas in the intestinal lymphoid tissue and also in the intestinal lamina propria. The diffuse lesions were characterized by severe and diffuse granulomatous enteritis and lymphadenitis that markedly altered the normal histological structure. According to the inflammatory cell type present in the infiltrate and the number of acid-fast bacilli (AFB), diffuse lesions were subdivided into diffuse lymphoplasmocytic or paucibacillary, diffuse intermediate and diffuse histiocytic or multibacillary lesions [61]. The different types correspond to the previously described latent (focal), patent (multifocal and diffuse) and apparently free (no lesions) forms of infection [62].

### 2.5. Immunohistochemistry (IHC) and Image Analysis

In order to validate ITLN2 as a biomarker of MAP infection, the number of cells expressing ITLN2 within the ICV of each animal was estimated by immunohistochemistry using a rabbit anti-ITLN2 polyclonal antibody. Briefly, formalin fixed paraffin embedded ICV samples were cut into 3-µm sections, mounted onto poly-L-lysine coated slides, dewaxed, rehydrated and rinsed with tap water at room temperature. Afterwards, epitope demasking was performed microwaving the slides in citric acid buffer pH 6.0 at maximum power for 5 min (four cycles, keeping the samples covered with buffer at all times) and ending with a 10 min incubation at room temperature (1.48 g citric acid (Merck KGaA, Darmstadt, Germany) in 500 mL distilled water). The sections were then washed with tap water. Then, slides were placed in a solution of methanol (VWR, Monroeville, PA, USA) with 3% hydrogen peroxide (Sigma-Aldrich, St. Louis, MO, USA) for 10 min at room temperature to block endogenous peroxidase activity and washed with tap water. Afterwards, the samples were treated to prevent primary antibody cross-reactivity with tissue constituents over a 20-min incubation period at room temperature, with 10% normal goat serum (Vector Laboratories) and 3% bovine serum albumin (BSA, Sigma-Aldrich, St. Louis, MO, USA). Then, sections were incubated overnight at 4 °C with a commercial rabbit polyclonal antibody anti-ITLN2 (Aviva Systems Biology, San Diego, CA, USA) at a 1/700 dilution. The immunogen used to produce this antibody is a synthetic peptide directed towards the middle region of human ITLN2, which has 100% amino acid sequence homology with bovine ITLN2. The predicted reactivity is 100%. Afterwards, samples were washed three times with Tris buffered saline 1× [TBS 1×, 5 mM Tris (Merck KGaA, Darmstadt, Germany)/HCl (Panreac Química, SLU, Barcelona, Spain) pH 7.6, 136 mM NaCl (Merck KGaA, Darstadt, Germany)] and then incubated for 30 min at room temperature with the anti-rabbit biotinilated secondary antibody produced in goat (Vector Laboratories, Burlingame, CA, USA) at 1/200 dilution. After that, samples were washed three times again with TBS 1× and incubated for 30 min at room temperature with Avidin-Biotin Complex (ABC kit Peroxidase Standard, Vector Laboratories). Finally, the sections were washed three times with TBS 1× and the signal was detected using DAB substrate [Citrate buffer, 0.2 M disodium hydrogen orthophosphate anhydro (Merck KGaA, Darmstadt, Germany)/0.1 M citric acid (Merck KGaA, Darmstadt, Germany) pH 6.4/a tablet of 10 mg 3,3′-Diaminobenzidine tetrahydrochloride (DAB, Sigma-Aldrich, St. Louis, MO, USA)/3% hydrogen peroxide (Sigma-Aldrich, St. Louis, MO, USA)] for 2 min. Samples were rinsed with tap water or for 5 min, before being placed in Mayer’s hematoxylin (MerckKGaA, Darmstadt, Germany) counterstain for 30 s. Finally, the slices were washed with tap water and mounted with DPX (MerckKGaA, Darmstadt, Germany). Negative controls (slides of the studied cows with omission of the primary antibody) were included.

Immunolabelled sections were observed with an Olympus BH-2 light microscope (Olympus, Tokyo, Japan) and photographed using an Olympus DP-12 digital camera (Olympus, Tokyo, Japan). Ten fields evenly distributed throughout all the mucosa section were examined at 400× magnification for each animal so that a total of 50 fields were examined for each histopathological type. The number of positive immunolabelled cells were manually counted in each selected field.

### 2.6. Statistical Analysis

Stained cell counts were submitted to the general linear model (GLM) procedure of the SAS statistical package (SAS Inc., Cary, NC, USA) to determine the statistical significance of differences in the mean number of labelled cells between the histopathological types. The multiple comparison adjusted Tukey’s test was applied to determine if there were statistically significant differences between geometric means. Statistical significance was accepted at *p* < 0.05. Data obtained from ITLN2 secreting cells quantification was analyzed using the Receiver operating characteristic (ROC) module of the MedCalc statistical software package version 19.6.4 (MedCalc software Ltd., Ostend, BE, https://www.medcalc.org; accessed on 31 March 2021), with confidence intervals stated at 95%. ROC curve analysis was used to determine the AUC (area under the curve), optimal cut off value, sensitivity and specificity of the method in order to make an approximation to its potential diagnostic value.

## 3. Results

### 3.1. Quantitative Immunohistochemical Evaluation of ITLN2 Secreting Cells within the Different PTB Histopathological Types

ITLN2 expression in ICV samples was investigated by quantitative immunohistochemistry to validate RNASeq results [31] and to evaluate its potential as a biomarker of disease progression.

**Figure 1:**
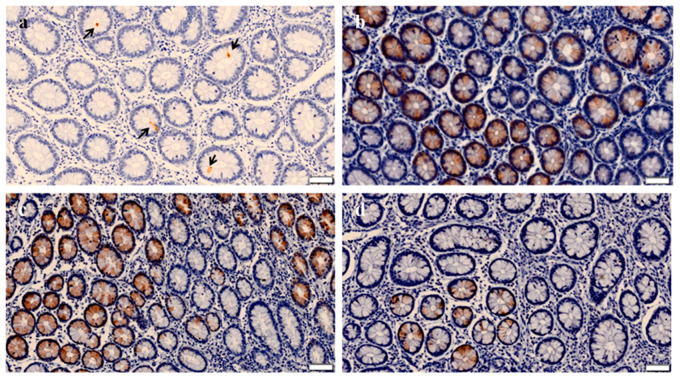
Immnohistochemical (IHC) analysis of ITLN2 in the ileocecal valve (ICV) of *Mycobacterium avium* subsp. *paratuberculosis*-infected and non-infected cows. Localization of ITLN2 biomarker was investigated by immunohistochemical analysis of ICV sections using a rabbit polyclonal anti-ITLN2 antibody. Representative IHC images of: (**a**) Control cow with no detected lesions; (**b**–**d**) cows with focal, multifocal and diffuse types of lesions in their gut tissues, respectively, are shown. The arrows indicate sites of specific antibody binding in the goblet and Paneth cells of the crypts of Lieberkühn. Note that in all cases, the staining (brown color) occurs at the bottom of the crypts of Lieberkühn, so it is only seen in the most circular sections, which are those that correspond to the deepest invagination. Bars represent 50 microns.

ITLN2 expression was validated on the entire range of lesions produced by MAP infection. As expected, ITLN2 was located exclusively in the cytoplasm of goblet and Paneth cells of the crypts of Lieberkühn (Figure 1). No specifically ITLN2 labelled cells were observed in the negative controls where IHC analysis of the ICV tissue sections was carried out with omission of the primary antibody.

**Figure 2:**
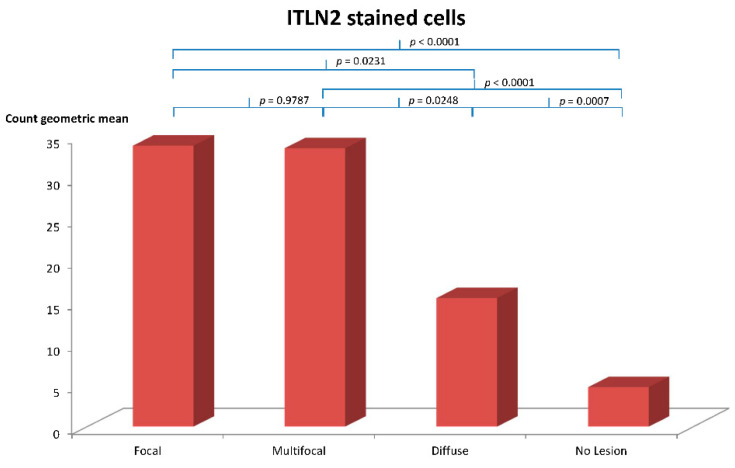
Quantification of positively immunolabelled Paneth and goblet cells in the ileocecal valve represented by the geometric mean of the number of ITLN2-stained cells per cow for each lesion type (focal, multifocal and diffuse) and the control with no lesions detected.

The mean number of immunostained cells was estimated per each individual cow (ten fields) and for each of the four established histopathological types (Table 2). The group of animals with focal lesions showed the highest mean number of ITLN2 immunostained cells per cow (116.9 ± 16.1), followed by the groups with multifocal (108.7 ± 19.9) and diffuse lesions (76.50 ± 13.8). The control group showed the lowest mean number of ITLN2 immunostained cells (41.0 ± 7.1). No significant differences were found between the geometric mean value of the ITLN2-labelled cells of the focal and multifocal lesion types (*p* = 0.9787), however, significant differences were found between the focal and the multifocal types with respect to the diffuse type (*p* = 0.0231 and *p* = 0.02481, respectively) and the control group (*p* < 0.0001 in both cases). Likewise, significant differences in the number of labelled cells were found between cows with diffuse lesions and the control cows (*p* = 0.0007) (Figure 2).

### 3.2. ROC Analysis

The diagnostic accuracy of the ITLN2 immunohistochemistry to discriminate between the different histopathological types and the control was estimated by ROC analysis (Table 3) as a preliminary assessment of its diagnostic potential. The best diagnostic performance was obtained for the multifocal type versus the control with an AUC value of 0.840 (95% confidence interval [CI]: 0.486–0.987), a sensitivity of 100% and a specificity of 80%. On the whole, the discriminatory power of the method between the different histopathological types and the control was good in all cases with AUC values in the range 0.8 ≤ AUC < 0.9, sensitivities between 90–100% and specificities between 60–80%. Specifically, the sensitivity and specificity of the method to detect cows with latent infections (focal lesions) were 100% and 80%, respectively, while to detect animals with patent infections (multifocal and diffuse types) the sensitivity was 90% and the specificity 80%. For overall detection of cows with any type of lesions the method has a sensitivity of 93.33% and a specificity of 80%. The best diagnostic value was obtained for detection of animals with focal and multifocal lesions (0.90 in both cases) and the worst for animals with diffuse lesions (0.80). The method had a good discriminatory power (0.8 ≤ AUC < 0.9) between the different histological types and the control group. These results showed that ITLN2 immunohistochemical quantification was a post-mortem tool, complementary to the histopathology, that could properly identify all forms of MAP infection.

## 4. Discussion

In this study, we investigated for the first time the potential of ITLN2 as a bovine biomarker of MAP infection. Immunohistochemical analysis of ICV sections showed that ITLN2 was located in the goblet and Paneth cells of the crypts of Lieberkühn, indicating that both types of cells were actively secreting ITLN2 (Figure 1). In spite of the small sample size used in this study (*n* = 5 for each histopathological type) significant differences in the geometric mean number of ITLN2 immunolabelled cells were found between the different histopathological types and the control group with no lesions detected beyond the standard *p* < 0.05 (focal vs. diffuse *p* = 0.0231; focal vs. control *p* < 0.0001; multifocal vs. diffuse *p* = 0.02481; multifocal vs. control *p* < 0.0001; diffuse vs. control *p* = 0.0007) indicating that ITLN2 is a good biomarker for the different stages of MAP infection. The level of secretion of ITLN2 was different for the different pathological types of PTB suggesting that ITLN2 plays an important role in the pathogenesis of the disease. Specifically, animals with focal lesions showed the highest mean number of immunostained cells secreting ITLN2 protein followed by animals with multifocal and diffuse lesions. These findings validate our previous RNA-Seq results which showed that bovine ITLN2 precursor gene was overexpressed in ICV of animals with focal (log2 fold-change = 10.6) and diffuse (log2 fold-change = 6.8) PTB-associated histological lesions compared to control animals without lesions [31]. Moreover, these results indicate that the amount of ITLN2 secreted by Paneth and goblet cells decreases as the disease progresses and the number of bacteria and the severity of clinical signs increase. It is known that Paneth cells help to maintain the balance of the gut microbiota by secreting anti-microbial peptides, cytokines and other trophic factors [65]. This intelectin could help to control the infection in its initial stages through the activation of the innate immune response, helping macrophages, one of the most abundant cells in focal lesions, to eliminate microorganisms. This function has already been suggested in previous studies for intelectin 1 (ITLN1) [37], a host defense lectin that assists in the phagocytosis of microorganisms [66], whose amino acid sequence has an identity of 91% with ITLN2 [67]. Furthermore, it is already known that ITLN2 plays a fundamental role in the innate immune response in infections, since it is overexpressed in mice infected with *Trichinella spiralis* and *Trichuris muris* [37,38], comparing in both cases with susceptible mice, or in zebrafish infected with *Staphyllococcus aureus* [68]. Studies carried out by Chen et al. [68] showed that the highest level of ITLN2 expression was found 10 h after bacterial infection. This increased expression of ITLN2 at early stages of infection has been observed in various species, including channel catfish, amphioxus and mouse [37,45,46,50]. In agreement with these findings, our RNA-Seq and immunohistochemical analysis of ICV samples showed that ITLN2 is highly overexpressed in MAP-infected animals with focal histological lesions (Table 1), which suggests that ITLN2 production is related to PTB forms of infection with no clinical signs, a predominant cell-mediated immune response and low bacterial load, supporting the above mentioned protective antibacterial role of ITLN2 in MAP latent infections of cows.

Although the multifocal and diffuse histopathological types also showed significant differences in the mean number of ITLN2 immunolabelled cells per cow with respect to the control type, the estimated means were lower (108.7 ± 19.9 and 76.50 ± 13.8, respectively). Thus, the multifocal type could be considered as an “intermediate” between the focal and diffuse lesion types since: (1) the number of ITLN2 secreting cells are lower than in the focal but higher than in the diffuse type (Table 3); (2) the amount of mycobacteria is greater but without reaching the levels of the diffuse type (Table 1) and (3) the clinical signs are already beginning to manifest in practically all cases and the cellular-type immune response continues to predominate over the humoral one (Table 1). In agreement with this, animals with diffuse lesions had lower numbers of ITLN2 immunolabelled cells (Table 2), higher bacterial loads and a predominant humoral immune response (Table 1).

As we mentioned above, an increase in ITLN2 secretion has also been observed in other pathologies indicating that this increase is not PTB specific suggesting that quantitative ITLN2 immunohistochemical analysis should be used in combination with standard morphologic evaluation for *post mortem* diagnosis of PTB.

Recent studies described the identification of four differentially expressed proteins (cathelicidin, haptoglobin, S100A8 and S100A9) by shotgun proteomics analysis of ileal tissues of sheep and their evaluation as potential biomarkers of the PTB paucibacillary histological type by immunohistochemistry [69]. MAP-free sheep tissues were negative to cathelicidin and haptoglobin and sparsely positive to S100A8 and S100A9 while PTB tissues were positive to all four proteins. The differential proteomic analysis combined with immunohistochemical validation highlighted several changes occurring in PTB tissues and provided novel molecular information on the distinct levels of tissue involvement that can be found within the asymptomatic, paucibacillary condition. Other studies have investigated the levels of inflammatory acute-phase proteins (haptoglobin and serum amyloid A) developed during the course of MAP infection in serum as potential serum markers for the identification of sub-clinically infected cows bearing pathological forms related to latency or resistance to the development of advanced clinical stages [70]. These authors observed a significant increase of the level of these proteins in infected animals, especially with lesions characterized by a low bacterial load and with predominance of a cell-mediated immune response.

Preliminary assessment of the diagnostic potential of the ITLN2 immunohistochemical quantification by ROC analysis confirmed its ability to discriminate between the different histopathological types and the control group. The diagnostic value of the quantitative ITLN2 immunohistochemical detection was very good in all cases (Table 3). Sensitivities for the detection of the different lesion types ranged from 90 to 100% indicating that ITLN2 immunohistochemistry can properly identify all forms of paratuberculosis. Specificity ranged from 60% for the detection of animals with diffuse lesions to 80% for the detection of animals with focal, multifocal and any type of lesions. This is mainly due to the fact that one of the control animals (ID13, Table 2) secreted high levels of ITLN2. Although this result might suggest that the method has some degree of non-specificity, it is possible that cow ID13 could represent a focal animal whose granuloma was not present in the area examined and therefore classification would require confirmation by examination of more tissue sections. It is worth mentioning that there is some degree of variability in the mean numbers of labelled cells obtained within each specific histopathological group, this might be due to the fact that the infectious status of each animal is different even within the same lesion type (Table 1). Our results validate the potential of ITLN2 as a biomarker for the different stages of MAP infection. Detection of bovine ITLN2 by immunohistochemical analysis of ICV tissue sections could constitute a good post-mortem tool, complementary to histopathology, which could improve detection of MAP infections. This is very important in the case of animals with latent infections, which may go unnoticed by conventional diagnostic tests, and for which, with our proposed method, the best diagnostic value was obtained (0.90). Detection of the presence of animals with latent infections on the farm is of great importance to establish an adequate management protocol and hygienic sanitary measures in order to reduce the incidence of PTB and for eradication campaigns.

## 5. Conclusions

This work provides the first characterization of ITLN2 expression in MAP-infected cows with different degrees of lesional severity in comparison to MAP-free animals. We proposed ITLN2 immunohistochemical analysis as a method to complement the conventional post-mortem histological methods used to diagnose PTB. Quantitative ITLN2 immunohistochemistry is able to detect animals with different degrees of lesion severity. We have found a significantly higher number of ITLN2 immunostained cells in animals with focal lesions, which is the predominant histological form in the initial stages of the disease, characterized by the absence of clinical signs, low bacterial load and a predominant cell-mediated immune response indicating that ITLN2 plays an important role in the progression and pathogenesis of the disease. These results validated ITLN2 as a biomarker of MAP infection, which is very important in the case of animals presenting latent forms of infection which tend to go unnoticed and therefore constitute a serious problem as these animals could spread MAP to the rest of the herd. Further studies in a larger and more heterogeneous sample are needed to confirm these conclusions.

## Figures and Tables

**Table 1:** MAP infection status of the 20 Holstein Frisian cows used in this study.

Animal ID	Age (years)		Diagnostic Method							
		Clinical Signs	Histological Classification	ZN	Infection Status	IDEXX ELISA	Real Time PCR		MAP Isolation	
							Feces	Tissues	Feces	Tissues
4	3.26	ND	No lesions	-	Non infected	NEG	NEG	NEG	NEG	NEG
13	0.81	ND	No lesions	-	Non infected	NEG	NEG	NEG	NEG	NEG
80	2.86	NO	No lesions	-	Non infected	NEG	NEG	NEG	NEG	NEG
94	2.70	NO	No lesions	-	Non infected	NEG	NEG	NEG	NEG	NEG
113	1.27	NO	No lesions	-	Non infected	NEG	NEG	NEG	NEG	NEG
8	4.71	NO	Focal	+	Infected	NEG	NEG	POS	NEG	POS
28	8.39	NO	Focal	-	Infected	NEG	POS	POS	NEG	POS
31	5.33	NO	Focal	-	Infected	NEG	NEG	POS	NEG	NEG
44	5.45	NO	Focal	-	Infected	NEG	NEG	NEG	NEG	POS
52	6.59	NO	Focal	-	Infected	POS	POS	POS	NEG	POS
11	7.31	NO	Multifocal	+	Infected	NEG	NEG	NEG	NEG	POS
46	2.75	YES	Multifocal	+	Infected	NEG	NEG	NEG	NEG	NEG
64	3.62	NO	Multifocal	+	Infected	POS	POS	POS	POS	POS
97	2.96	YES	Multifocal	++	Infected	POS	POS	POS	NEG	POS
110	4.03	NO	Multifocal	-	Infected	NEG	NEG	POS	NEG	POS
26	5.46	NO	D. intermediate	+	Infected	POS	POS	POS	NEG	POS
30	4.14	YES	D.intermediate	+	Infected	NEG	NEG	NEG	NEG	NEG
68	6.01	YES	D. intermediate	++	Infected	POS	POS	POS	POS	POS
88	2.92	YES	D. histiocytic	+++	Infected	POS	POS	POS	NEG	POS
103	10.39	YES	D. histiocytic	+	Infected	POS	POS	POS	NEG	POS

ID, identification number of each cow; ND: no determined; NO: absence of clinical signs associated to paratuberculosis; YES: presence of at least one of the clinical signs of the disease (mainly diarrhea, progressive weight loss and decreased milk production); NEG: negative; POS: positive; ZN, Ziehl-Neelsen stain; -: no acid-fast bacilli (AFB), +: low number of AFB; ++: moderate number of AFB; +++: high number of AFB; IDEXX ELISA, ELISA to determine the presence of anti-MAP antibodies; D., Diffuse. Animals with focal histological lesions are considered to have a latent infection while animals with multifocal and diffuse lesions (diffuse intermediate and diffuse histiocytic or multibacillary) are considered to have patent infections.

**Table 2:** Quantification of ITLN2-labelled cells present in the ICV samples of control animals with no lesions or animals with focal, multifocal and diffuse histological lesions.

Animal ID	Histopathological Classification	Infection Status	Mean Number	Mean Value
			per Cow *	per Type **
4	No lesions	Non-infected	0.1 ± 0.0	
13	No lesions	Non-infected	184.5 ± 82.9	
80	No lesions	Non-infected	2.2 ± 4.7	41.0 ± 7.1
94	No lesions	Non-infected	16.7 ± 17.9	
113	No lesions	Non-infected	1.7 ± 4.4	
8	Focal	Infected	80.7 ± 89.6	
28	Focal	Infected	174.9 ± 116.4	
31	Focal	Infected	146.6 ± 71.5	116.9 ± 16.1
44	Focal	Infected	30.3 ± 67.8	
52	Focal	Infected	152.1 ± 153.4	
11	Multifocal	Infected	253.3 ± 210.8	
46	Multifocal	Infected	28.5 ± 38.9	
64	Multifocal	Infected	154.3 ± 122.3	108.7 ± 19.9
97	Multifocal	Infected	37.8 ± 45.5	
110	Multifocal	Infected	69.4 ± 67.1	
26	Diffuse intermediate	Infected	34.6 ± 40.3	
30	Diffuse intermediate	Infected	15.8 ± 26.7	
68	Diffuse intermediate	Infected	46.0 ± 66.0	76.50 ± 13.8
88	Diffuse histiocytic	Infected	90.6 ± 107.5	
103	Diffuse histiocytic	Infected	195.7 ± 103.5	

ID, identification number of each cow; * The number indicates the mean number of ITLN2-labelled cells in the 10 fields counted per cow; ** The number represents the mean value ± standard error of the mean per lesion type (5 × 10 fields were counted per lesion type). The sample unit used in this analysis is the field.

**Table 3:** Diagnostic performance of bovine intelectin 2 immunohistochemical quantification for diagnosis of cattle with different types of PTB-associated histological lesions in their gut tissues.

ROC Analysis	AUC	*p* Value	CUT OFF	SE (%)	SP (%)	DV
Focal vs. Control	0.800	0.134	>167	100.00	80.00	0.90
Multifocal vs. Control	0.840	0.039	>167	100.00	80.00	0.90
Diffuse vs. Control	0.800	0.073	>22	100.00	60.00	0.80
Patent vs. Control	0.820	0.046	>167	90.00	80.00	0.85
Lesions vs. Control	0.813	0.069	>167	93.33	80.00	0.86

ROC, receiver operating characteristic, ROC analysis was used to determine the diagnostic performance of the method; AUC, area under the curve; *p*-value, it is the *p*-value of the AUC area, indicates whether the discrimination between animals with focal, multifocal, diffuse or any type of lesions and controls is significant; The cut-off point is expressed as the total number of ITLN2 positive cells per cow; SE, sensitivity; SP, specificity; DV, diagnostic value (semi-sum of the sensitivity and specificity); VS., versus. C, control type consisting of five animals with no lesions detected; The number of animals analyzed in the focal, multifocal and diffuse types were also five (*n* = 5 per group). The patent type includes the five animals with multifocal lesions and the five animals with diffuse lesions (*n* = 10). The discriminatory power of each biomarker to discern between the different histopathological types and the control type was classified as follows: AUC values ≥ 0.9 were considered to have excellent discriminatory power; 0.8 ≤ AUC < 0.9 good discriminatory power; 0.7 ≤ AUC < 0.8 fair discriminatory power; and AUC < 0.7, poor discriminatory [63,64].

## Data Availability

Not applicable.
